# Health Status, Behaviors, and Beliefs of Health Sciences Students and Staff at Kuwait University: Toward Maximizing the Health of Future Health Professionals and Their Patients

**DOI:** 10.3390/ijerph17238776

**Published:** 2020-11-26

**Authors:** Nowall Al-Sayegh, Khazna Al-Enezi, Mohammed Nadar, Elizabeth Dean

**Affiliations:** 1 Department of Physical Therapy, Faculty of Allied Health Sciences, Kuwait University, Safat 12037, Kuwait; 2 Department of Medicine, Faculty of Medicine, Kuwait University, Safat 12037, Kuwait; khaznah.alenezi@ku.edu.kw; 3 Department of Occupational Therapy, Faculty of Allied Health Sciences, Kuwait University, Safat 12037, Kuwait; ot_nadar@hsc.edu.kw; 4 Department of Physical Therapy, Faculty of Medicine, University of British Columbia, 212 Friedman Building, 2177 Wesbrook Mall, Vancouver, BC V6T 1Z3, Canada; elizabeth.dean@ubc.ca

**Keywords:** health promotion, health professionals, lifestyle behavior, health sciences students, Kuwait

## Abstract

Health professionals who engage in healthy lifestyle behaviors are more likely to promote their patients’ health. We evaluated health status, behaviors, and beliefs of students (future health professionals) and staff in four health sciences faculties, Kuwait University. In total, 600 students and 231 staff participated in this descriptive cross-sectional study. Questionnaire surveys were used to evaluate lifestyle-related practices and participants’ beliefs about these practices, in addition to health-related objective measures, e.g., heart rate, blood pressure, and body mass index. Overweight/obesity was prevalent among the participants (staff, 68.7%, students, 48.1%; *p* < 0.001); 57% of staff had suboptimal resting blood pressures. About half of the participants reported being moderately physically active (staff, 44.8%, students, 52.6%; *p* < 0.05), and most reported moderate/high stress (staff, 88.8%, students, 90.9%; *p* > 0.05). Only 25.1% of staff and 27.9% of students reported at least 8 h sleep nightly (*p* > 0.05). Staff reported healthier dietary practices than students (*p*-value range < 0.001–0.02). Overall, the participants had sub-optimal health indices. A marked gap existed between participants’ beliefs about healthy lifestyle practices and their actual health status. Healthy lifestyle programs are needed on campus with respect to diet, exercise, and stress management. As emerging health professionals, students in health sciences faculties, Kuwait University, need exposure to a health-promoting environment including healthy staff as role models.

## 1. Introduction

Health is a function of personal and environmental factors that reflects physical, mental, and social well-being [1,2]. Health professionals have a responsibility to serve as role models for their patients, given they can influence the health status of thousands of individuals and their families over their careers [3,4,5,6,7,8]. Despite this, their poor personal health practices [9] tend to resemble those of the general population rather than being superior, as might be predicted by virtue of their professional career choice [10]. Health professionals who have good physical health and healthy lifestyle practices have been reported to be more likely to engage in health promotion with patients, and their advice is perceived as being more credible [11]. Accordingly, the values of healthy lifestyle practices [12,13,14] should be instilled in health sciences students as central to their professional socialization, for the benefit of their future patients as well as to maximize their overall health as students.

University students report barriers to healthy living practices including exercise, and this appears to be prevalent worldwide [15,16,17,18,19,20,21,22,23,24]. Suboptimal health indices of health sciences university students do not bode well for their serving as future practitioners and health role models. Although in studies related to the characteristics and health promotion practices of healthcare practitioners it is not known whether these professionals were active as students, the literature is clear that practitioners with poor practices are less likely to counsel patients [25,26,27]. Nonetheless, physicians’ health practices are key predictors of their engaging in health promotion counseling clinically [7,11]. 

Preliminary findings based on objective measures and lifestyle-related practices suggested that health sciences students attending Kuwait University have poor indices of health [28]. The purpose of the present study was to extend these findings to a large cohort of students, as emerging health professionals, and staff. Our primary objective was to profile the health status, behaviors, and beliefs of students and staff who were studying or working across four health sciences faculties at Kuwait University. Given gender intersects with multiple aspects in life and directly impacts the determinants of health [29,30,31], a secondary objective was to compare genders with respect to the variables of interest. 

Based on this baseline, our intent is to collect longitudinal data on the health status of students and staff annually as a component of our ‘culture of health’ campaign at the health sciences campus of Kuwait University. We conceptualized a ‘culture of health’ as an environment inclusive of the physical space, resources, and people—i.e., students and academic and non-academic staff—and in which health and health-related lifestyle practices are promoted through our health assessment and education initiatives. Non-academic staff were included given their interface and interactions with students and staff through their principal administrative and teaching support roles. Conducting detailed annual health assessments will enable our team to provide expert individualized health recommendations and follow-up over successive years, as well as provide means of informing students and staff about health education initiatives and services and health promotion programs on campus. These initiatives are being informed and developed based on our data. Our eventual aim is to construct an exemplary ‘culture of health’ for university campuses responsible for educating health professionals. In addition, given that health professionals are often subject to the dire burn-out phenomenon [32,33], they are more likely to experience optimal health throughout their careers with healthy lifestyle awareness and practices, as well as promote these practices in their patients. 

## 2. Materials and Methods 

### 2.1. Participants

We recruited students across all four years of their programs and staff volunteers from the four health science faculties at Kuwait University, namely, allied health professions, dentistry, medicine, and pharmacy, during one academic year (2016–2017) with recruitment notices and through deans and department heads, whom we invited to support this campus-wide health initiative. All enrolled students and full-time staff were eligible to participate in the study. 

This study was a descriptive cross-sectional investigation with the objective of serving as a baseline for a longitudinal study over successive years of data collection. Staff members eligible to participate included academic and non-academic support staff. Ethics approval was obtained from the ethical review board of Kuwait University. All participants provided signed consent prior to data collection. They were assured complete confidentiality. Their names and contact information were only known to the primary investigator. The study was approved by the Institutional Review Board at Kuwait University. All participants provided their informed consent prior to participation in the study. Report of data analysis undertaken and a copy of the survey instrument are available from the corresponding author on reasonable request.

### 2.2. Questionnaire and Objective Measures

A questionnaire which was administered by trained data collectors consisted of four sections: (1) personal demographic data; (2) a checklist of the health status; (3) lifestyle-related practices and attributes, including physical activity habits, diet, smoking, stress level, and sleep; and (4) lifestyle-related beliefs about these practices and attributes. The validity of the questionnaire was previously established [30,34]. The objective health measures recorded were heart rate, blood pressure, height, weight, hip circumference, and random blood glucose. Based on height and weight measurements, body mass index (BMI) was calculated (kg·m^−2^). Waist/height ratio and waist/hip ratio were also calculated. All the objective health measures were assessed based on standardized methods and procedures, with established psychometric properties [28]. We used cut points from multiple reputable sources to establish whether participants’ health parameters were categorized as within healthy ranges or not (see Appendix A for more details).

### 2.3. Personal and Group Strategies

Consistent with our construct of growing a ‘culture of health’ on campus and based on health assessments of the participants, we provided targeted education tailored to their health outcomes and guidelines to achieve healthy measurements as well as guidelines for exercise and physical activity. We explained how to calculate and analyze measurements such as waist-to-hip and waist- to-height ratios and body mass index. For some participants, we addressed musculoskeletal issues and how to manage them, especially during physical activity, and issues related to sedentary behavior and how to break up long periods of desk sitting. Participants were also encouraged to identify facilitators and barriers to healthy lifestyle practices including exercise, nutrition, and sleep and were guided in terms of setting realistic and measurable goals. In terms of group strategies, we encouraged participants, particularly the students, to create family and friendship networks with those who shared similar goals, so they could exercise together and support each other. We discussed with participants about the negative effects of prolonged sitting and insufficient sleep and proposed strategies for stress management. We believed it was important to stress this to staff and students. Future studies will report on the effect of these educational initiatives within our campus ‘culture of health’. 

### 2.4. Statistical Analysis 

The data were analyzed with SPSS version 24 (IBM, Chicago, IL, USA). Descriptive statistics were used to characterize the data of participating students and staff. In addition, Chi-square and Fisher’s exact tests were used to compare the findings for students versus those for staff members and the findings for men versus those for women. 

## 3. Results

The sample consisted of 831 volunteer participants, with greater participation of women (*n* = 648) than men (*n* = 183) and of students (600) than staff members (231). Table 1 shows summary statistics for age and sex distribution for the students and staff groups.

When comparing the objective indices of health, overweight/obesity (BMI ≥ 25 kg·m^−2^) was observed in both the staff and the student cohorts (68.7% and 48.1% respectively, *p* < 0.001). In addition, staff members had higher resting blood pressures (in excess of 120/90 mm Hg). Abnormal random blood sugars (>11.1 mmol/L) suggestive of pre-diabetes was unusual in both sexes, with only 1% occurrence in men and <1% in women, across the two cohorts. 

Objective and behavioral characteristics of lifestyle practices and attributes according to participant role (student or staff member) are shown in Table 2. Staff members tended to be heavier than students, with waist/height and waist/hip ratios in the unhealthy ranges. These findings were proportionate to their observed higher resting blood pressures. Although similar to sex differences, pre-diabetic levels of random blood glucose were observed infrequently: students had less than 1% occurrence rate, and staff exceeded 1% occurrence. This low prevalence of pre-diabetes may be due to measuring random blood glucose rather than fasting glucose. About 94.3% of students had never smoked compared with staff (85.3%). The current smoking rates were reportedly higher in staff than in students (9.3% vs. 5.0%). Most participants in both groups reported experiencing moderate/high levels of stress and slept less than 8 h nightly. Role differences (student vs. staff) were also observed for dietary patterns. Staff members’ eating practices were generally healthier than those of students. They consumed more of the recommended daily servings of fruit and vegetables and consumed the lower recommended servings of animal protein except for fish, for which at least two servings a week are recommended. Egg consumption was comparable for the two groups and within the guideline of one every other day. Students reported consuming more carbonated drinks (*p* ≤ 0.001) and missing breakfast more frequently (*p* ≤ 0.001) than staff.

Objective and behavioral characteristics (lifestyle practices) and attributes for men and women in the sample appear in Table 3. Women tended to be healthier than men in terms of blood pressure, BMI, waist/height and waist/hip ratios. About half of each group reported a weekly level of physical activity below the recommended one, although men were generally more active than women. In addition, 20.9% of men reported being current smokers, compared with only 2.0% of women. Women reported greater life stress than men. Most men (78.7%) slept less than 8 h a night compared with women (71.2%). Sex differences were observed for dietary patterns as well; women ate fewer eggs and animal protein than men, except for fish which women reported consuming more frequently. About one-quarter of the participants did not eat breakfast daily. 

When asked about the degree to which they believed that the lifestyle-related factors of physical activity, nutrition, smoking, and stress impact general health, 97.7% of staff and 96.2% of students agreed/strongly agreed that all four lifestyle practices impacted health (Table 4). However, these beliefs and participants’ actual measures of health status were discrepant.

## 4. Discussion

Given health professionals who engage in healthy lifestyle practices themselves are more likely to engage in health promotion with patients clinically, we chose to investigate the health of health sciences students—i.e., emerging health professionals—and staff, given the latter are central to students’ learning environment. Based on our questionnaire and objective measures and indexes of health status, our findings are discussed in terms of comparisons between students and staff and between women and men. Then, we discuss the overall implications of our findings and study strengths and limitations. 

### 4.1. Comparison of Students and Staff

Based on 831 participants, our findings confirm the presence of sub-optimal health practices and non-communicable disease risk factors for students and staff studying and working at the health sciences campus, Kuwait University. The objective measures of health status were generally superior (i.e., a higher proportion within a healthy range) for students compared with staff (specifically, BMI, resting systolic and diastolic blood pressures, waist/height ratio, and waist/hip ratio). This could reflect the fact that staff members were on average 20 years older, thus manifesting signs associated with living for multiple years with adverse health practices. With respect to behavioral practices and attributes however, staff members generally reported healthier practices than students, with the exception of smoking history. Specifically, staff consumed more of the recommended types of foods and servings for optimal health than students. This may reflect a greater awareness of staff being middle-aged and the need to pay closer attention to healthy lifestyle practices. Staff members have a pivotal role in the professional socialization of students in health sciences education programs, thus the health attributes and practices they model to students are critical [4,5]. The attention of students and staff to their health and lifestyle practices is important not only for their overall personal health and wellbeing, but also to maximize wellbeing, productivity, and effectiveness in their respective demanding roles. 

Consistent with the literature [9], the lifestyle-related practices of both students and staff were not congruent with their beliefs, that in our study happen to be well supported by evidence, i.e., the importance of physical activity, healthy diet, healthy weight, not smoking, sleep, and manageable stress on overall health (Table 4). The lifestyle-related beliefs of our participants were similar to those reported recently for adult community dwellers living in Kuwait who reported beliefs about the benefits of healthy living practices and attributes to health, such as not smoking, healthy nutrition, regular physical activity, manageable stress, and optimal sleep, than they actually practiced [34]. Further, given the variations observed between students and staff, health promotion campaigns in university settings such as Kuwait University need to consider people’s ages and life cycle stage as core factors when targeting and tailoring such campaigns. 

Our findings are consistent with other studies of the health of university students in the Middle East. Previous studies tended to examine isolated lifestyle practices, whereas our study was comprehensive in terms of lifestyle practices and attributes examined. Commonly, previous studies focused on physical activity, nutrition, or both [35,36,37]. Nasser and colleagues focused on smoking cigarettes and waterpipes and reported that these practices were alarmingly high in university students in a cross section of Arab countries [38]. Our findings showed 20.9% of the men and only 2% of women reported being current smokers. The gender differential may be explained by cultural issues and potentially under-reporting in women. An initiative to evaluate the lifestyle practices of Iranian university students, including tobacco and alcohol use and sleep, has been reported with the potential for long-term tracking purposes [39]. Finally, studies of university students’ mental health are also emerging, reporting untoward levels of anxiety and depression [40,41]. Our cohort reported high levels of stress for both sexes, which can contribute to mental ill health. 

### 4.2. Comparison of Female and Male Participants

Health promotion research indicates that gender is a critical factor when designing effective health promotion programs, as gender impacts other determinants of health, such as health contexts and how women and men may take care of their health and, in turn, respond to health promotion efforts [27,28,29]. Compared with men, women in our sample tended to have superior health attributes and practices. Except for resting heart rate, female participants were, proportionately, more within healthy ranges than male participants for BMI, systolic and diastolic blood pressures, and waist/height and waist/hip ratios. Regarding lifestyle-related practices, women were more often in the healthy ranges for never having smoked, fruit and vegetable consumption, and lower animal protein consumption. Both sexes consumed less than the recommended daily servings of fruit and vegetables and were high consumers of fast food. Based on the well-established health protective effects of vegetable sources of nutrients, increasing daily fruit and vegetable and decreasing fast food consumption [42] warrant greater attention in student health education. With respect to previous studies which reported women are more health-conscious, the health attributes and practices of women in our study were not exceptionally superior to those of the men, which is somewhat inconsistent with the literature on sex differences [30,31,34,43,44].

When cohort role, either student or staff, was considered, discordance was observed between lifestyle-related practices/attributes of women and men, as well as their beliefs about the importance of not smoking, physical activity, healthy diet, sleep hygiene, and manageable stress, to overall health. Although their beliefs were consistent with the prevailing evidence for promoting maximal health, these beliefs failed to translate into actual lifestyle practices. 

The social structure within which people live shapes their health beliefs and practices in various ways, including the socialization of women and men [29,30,31]. Therefore, gender warrants being addressed and integrated into targeted health promotion strategies, which is supported by our findings. Public health promotion initiatives that acknowledge gender disparity and are sensitive to gender distinctions within a culture are more effective [29,30,31]. Thus, health education frameworks that draw attention to gender disparity may more effectively increase awareness about healthy lifestyle choices, thus being more cost-effective as well.

### 4.3. Implications

The sub-optimal health status of the health science students in our cohort, which is not atypical for university students, is concerning for several reasons. First, there has long been a call for action to address unhealthy lifestyle practices of people living in Kuwait, due to the increased prevalence of non-communicable diseases and their risk factors in the country [45,46,47,48]. Second, given their poor health practices which jeopardize their health and wellbeing, the people of Kuwait need to be able to look up to their health professionals as role models and to access health-related guidance, being these professionals perceived as credible. Healthy health professionals make strong impressions on their patients in terms of their practicing healthy living recommendations [8]. Lack of time, resources, attainable exercise facilities on campus, and the discipline to pursue these healthy lifestyle behaviors is commonly cited by university students as limiting their capacity for healthier lifestyle practices [28,49]. Thus, through our ‘culture of health’ initiative at Kuwait University, we are developing strategies to encourage healthy lifestyle behaviors within the academic community, with a focus on students as emerging health professionals. 

Our ‘culture of health’ initiative on the health sciences campuses is innovative and, to the best of our knowledge, has not been replicated elsewhere. It was developed by the Department of Physical Therapy and is led by both faculty and students. Student engagement promotes buy-in from other students on campus, where students teach other students with a view to promote physical and mental health and wellbeing [50,51]. 

In designing health promotion programs in general, the context of the potential participants needs to be considered foremost for programs to be sustained over the life span, principally after graduation for students. Kuwait is an oil-rich, Arab Muslim country that has undergone rapid economic development with the discovery of oil in the 1930s [45,46,47,48]. Commensurate with globalization, access to unhealthy lifestyle choices has escalated, such that the people of Kuwait, adults and children, generally have non-communicable disease risk factors, including those for heart disease, type 2 diabetes mellitus, and obesity, or their manifestations [52]. These trends of epidemic proportions are consistent with the global trends [46,47]; hence, multiple calls to action at national and international levels are required.

Several other contextual factors can pose limitations to designing health promotion programs in Kuwait. Although Kuwait is considered a moderate Islamic country in terms of religious and civil liberties, there are moderate gender restrictions that are imposed by tradition rather than religion. Women are less likely to be on their own in public, thus are less likely to exercise outdoors compared with men, e.g., they do not swim publicly on beaches. The neighborhoods in general are less walker-friendly (e.g., no sidewalks, uneven pavement, and traffic). Climatic considerations also limit physical activity and exercise alternatives for several months of the year. From May to October, Kuwait experiences extreme temperatures that often exceed 40 °C. Under these conditions, heat-generating outdoor physical activity is dangerous, contributing quickly to dehydration and sunburn with direct exposure. 

### 4.4. Strengths and Limitations

To our knowledge, this is the first large cohort study of university students as future health professionals and staff as central student role models in the students’ academic socialization environment. Also, it is the first of its kind with a defined long-range goal of creating a ‘culture of health’ on a health sciences campus, with a view of creating exemplary health professionals who are models of health themselves, value health, and are committed to pursuing it. 

With respect to limitations, our sample consisted of volunteers, hence, the potential for volunteer bias. This convenience sampling might have been composed of health-conscious individuals who were more motivated to participate. Alternatively, they might have self-selected to participate because of their awareness for needing assistance to improve their health. Because our findings are limited to Kuwait, replication and extension studies are needed in other geographic regions. 

## 5. Conclusions

For health professionals to serve as credible role models for the patients they serve, they need to have exemplary lifestyle practices themselves. This process should be central in their professional socialization during their university studies. Our findings highlight the sub-optimal health characteristics and attributes of students—our future health professionals—and staff—who are an integral component of the academic professional socialization environment—at Kuwait University. Specifically, the participants exhibited unhealthy attributes and lifestyle practices and marked discordance between these practices and their largely evidence-supported beliefs about the benefits of healthy lifestyle practices. With the elucidation of this gap between lifestyle practices and beliefs, our findings help inform personal and group strategies to address sub-optimal health behaviors and attributes in the students and staff and also help align students’ and staff’s generally evidence-supported health beliefs with their lifestyle-related choices and practices. 

Students and staff are being urged to participate in annual health assessments and take advantage of individualized health coaching, advice, and follow-up. In this way, the baseline of student and staff health will be expanded and evaluated in terms of change over years in students’ academic program or staff members’ years of employment. Finally, studies are being designed to elucidate the facilitators for and barriers to healthy living practices for students and staff members within the university setting, to further refine campus health education campaigns. Supporting the health of a campus community is particularly important at this time, given the global coronavirus disease 2019 (COVID-19) pandemic. Given the strong association between COVID-19 mortality and multimorbidity, it is imperative that risk factors for non-communicable diseases (e.g., high blood pressure, obesity, and abnormal blood sugars) are controlled within our emerging health professionals, so to further protect, in turn, the patients they influence.

## Figures and Tables

**Table 1:** Mean ages (standard deviations) of the participants.

Age (Years)	*n*	Mean	(SD)	*p*-Value
All participants	831	25.7	(10.7)	
Students and Staff				
Students	600	20.3	(2.6)	≤0.001
Staff	231	39.9	(11.0)	
Sex (Students and Staff pooled)				
Men	183	29.4	(13.7)	≤0.001
Women	648	24.7	(9.5)	
Sex (Students only)				
Men	116	20.8	(3.6)	≤0.001
Women	484	20.2	(2.2)	
Sex (Staff only)				
Men	65	44.7	(11.6)	0.013
Women	162	37.9	(10.1)	

Grouped by students, staff, and sex. Numbers do not add up to the total due to missing values.

**Table 2:** Objective and behavioral characteristics of staff vs. students.

	Staff		Students		*p*-Value
	*n* = 231		*n* = 600		
	*n*	(%)	*n*	(%)	
Body mass index (kg·m^−2^)					
Healthy (18.5 to <25)	72	(31.3)	314	(51.9)	≤0.001
Unhealthy (≥25)	158	(68.7)	291	(48.1)	
Heart rate (bpm)					
Healthy (60–80)	109	(47.4)	160	(26.5)	≤0.001
Unhealthy (<60 if symptomatic or >80)	121	(52.6)	444	(73.5)	
Systolic blood pressure (mm Hg)					
Healthy (≤120)	99	(43.0)	341	(56.6)	≤0.001
Unhealthy (>120)	131	(57.0)	262	(43.4)	
Diastolic blood pressure (mm Hg)					
Healthy (≤80)	154	(67.0)	507	(84.1)	≤0.001
Unhealthy (>80)	76	(33.0)	96	(15.9)	
Random blood glucose (mmol/L)					
Healthy (≤11.1)	227	(98.7)	600	(99.5)	0.355
Unhealthy (>11.1)	3	(1.3)	3	(0.5)	
Waist: height ratio					
Healthy (<0.5)	96	(41.9)	459	(76.0)	≤0.001
Unhealthy (≥0.5)	133	(58.1)	145	(24.0)	
Waist: hip ratio					
Healthy (men < 0.9, women < 0.85)	172	(75.1)	584	(96.7)	≤0.001
Unhealthy (men ≥ 0.9, women ≥ 0.85)	57	(24.9)	20	(3.3)	
Moderately physically active (at least 3.5 h/week)					
Yes	103	(44.8)	318	(52.6)	0.042
No	127	(55.2)	286	(47.4)	
Smoking status					
Never smoke	192	(85.3)	563	(94.3)	≤0.001
Past smoker	12	(5.3)	4	(0.7)	
Current smoker	21	(9.3)	30	(5.0)	
Self-reported life stress					
Low	25	(11.2)	54	(9.1)	0.369
Moderate/high	198	(88.8)	538	(90.9)	
Sleep quantity (hours/night)					
≥8	53	(25.1)	148	(27.9)	0.446
<8	158	(74.9)	383	(72.1)	
	*n*	(%)	*n*	(%)	
Fresh fruit (standard servings/day)					
≥5	24	(11.6)	27	(5.3)	0.003
<5	183	(88.4)	485	(94.7)	
Fresh vegetables (standard servings/day)					
≥5	19	(9.0)	22	(4.2)	0.011
<5	192	(91.0)	498	(95.8)	
Beef or sheep (standard servings/week)					
<2	112	(49.6)	238	(40.4)	0.018
≥2	114	(50.4)	351	(59.6)	
Meat products (standard servings/week)					
<2	169	(75.8)	263	(45.0)	≤0.001
≥2	54	(24.2)	322	(55.0)	
Red meat (beef or sheep/ meat product)					
<2	93	(41.3)	145	(24.7)	≤0.001
≥2	132	(58.7)	441	(75.3)	
Fish (standard servings/week)					
≥2	90	(39.6)	114	(19.6)	≤0.001
<2	137	(60.4)	469	(80.4)	
Eggs (number/week)					
<4	185	(81.5)	483	(82.0)	0.867
≥4	42	(18.5)	106	(18.0)	
Fast food frequency/week)					
None	48	(21.2)	46	(7.7)	≤0.001
≥1	178	(78.8)	549	(92.3)	
Sweets (standard servings/week)					
≤3	226	(100.0)	587	(99.7)	0.999
>3	0	(0.0)	2	(0.3)	
Soft drinks (cans/week)					
None	107	(47.1)	221	(38.2)	0.020
≥1	120	(52.9)	358	(61.8)	
Breakfast daily					
Yes	189	(87.5)	400	(69.8)	≤0.001
No	27	(12.5)	173	(30.2)	

Frequency counts shown as *n* (%); *p*-values were generated using Chi-square test except for random blood glucose (mmol/L) and sweets (standard servings/week), for which Fisher’s exact test was used due to the small numbers.

**Table 3:** Objective and behavioral characteristics of males vs. females.

	Men		Women		*p*-Value
	*n* = 186		*n* = 655		
	*n*	(%)	*n*	(%)	
Body mass index (kg·m^−2^)					
Healthy (18.5 to <25)	61	(32.8)	325	(49.8)	≤0.001
Unhealthy (≥25)	125	(67.2)	328	(50.2)	
Heart rate (bpm)					
Healthy (60–80)	96	(51.9)	175	(26.8)	≤0.001
Unhealthy (<60 if symptomatic, or >80)	89	(48.1)	478	(73.2)	
Systolic blood pressure (mm Hg)					
Healthy (≤120)	39	(21.1)	405	(62.1)	≤0.001
Unhealthy (>120)	146	(78.9)	247	(37.9)	
Diastolic blood pressure (mm Hg)					
Healthy (≤80)	116	(62.7)	549	(84.2)	≤0.001
Unhealthy (>80)	69	(37.3)	103	(15.8)	
Random blood glucose (mmol/L)					
Healthy (≤11.1)	183	(98.9)	648	(99.4)	0.619
Unhealthy (>11.1)	2	(1.1)	4	(0.6)	
Waist/height ratio					
Healthy (<0.5)	96	(51.9)	460	(70.6)	≤0.001
Unhealthy (≥0.5)	89	(48.1)	192	(29.4)	
Waist/hip ratio					
Healthy (men <0.9, women <0.85)	143	(77.3)	617	(94.6)	≤0.001
Unhealthy (men ≥0.9, women ≥0.85)	42	(22.7)	35	(5.4)	
Moderately physically active (at least 3.5 h/week)					
Yes	111	(59.7)	304	(46.6)	0.002
No	75	(40.3)	348	(53.4)	
Smoking status					
Never smoke	131	(72.0)	628	(97.5)	≤0.001
Past smoker	13	(7.1)	3	(0.5)	
Current smoker	38	(20.9)	13	(2.0)	
Self-reported life stress					
Low	28	(15.3)	52	(8.2)	0.004
Moderate/high	155	(84.7)	584	(91.8)	
Sleep quantity (hours/night)					
≥8	36	(21.3)	167	(28.8)	0.052
<8	133	(78.7)	412	(71.2)	
Fresh fruit (standard servings/day)					
≥5	16	(10.1)	35	(6.2)	0.087
<5	142	(89.9)	531	(93.8)	
Fresh vegetables (standard servings/day)					
≥5	5	(3.1)	36	(6.3)	0.131
<5	154	(96.9)	540	(93.8)	
Beef or sheep (standard servings/week)					
<2	45	(25.1)	306	(48.0)	≤0.001
≥2	134	(74.9)	332	(52.0)	
Meat products (standard servings/week)					
<2	83	(47.2)	350	(55.2)	0.058
≥2	93	(52.8)	284	(44.8)	
Red meat (beef or sheep/ meat products) (servings/week)					
<2	31	(17.3)	208	(32.8)	≤0.001
≥2	148	(82.7)	426	(67.2)	
Fish (standard servings/week)					
≥2	58	(32.4)	148	(23.3)	0.014
<2	121	(67.6)	486	(76.7)	
Eggs (number/week)					
<4	129	(71.3)	543	(85.0)	≤0.001
≥4	52	(28.7)	96	(15.0)	
Fast food (frequency/week)					
None	23	(12.4)	72	(11.3)	0.657
≥1	162	(87.6)	568	(88.8)	
Sweets (standard servings/week)					
≤3	182	(100.0)	635	(99.7)	0.999
>3	0	(0.0)	2	(0.3)	
Soft drinks (cans/week)					
None	70	(38.3)	262	(41.8)	0.392
≥1	113	(61.7)	365	(58.2)	
Breakfast daily					
Yes	131	(74.9)	618	(74.4)	0.910
No	44	(25.1)	158	(25.6)	

Frequency counts shown at *n* (%); *p*-values were generated using Chi-square test except for random blood glucose (mmol/L) and sweets (standard servings/week) for which Fisher’s exact test was used due to the small numbers.

**Table 4:** Participants beliefs about the relationship between four lifestyle-related behaviors/attributes and general health.

Behavior/Attribute	Participants	*n*	Strongly Agree	Agree	Not Sure	Disagree	Strongly Disagree
			*n* (%)	*n* (%)	*n* (%)	*n* (%)	*n* (%)
Do you believe that physical activity makes a difference for a person’s health overall?	Students	578	442 (76.5)	115 (19.9)	15 (2.6)	1 (0.2)	5 (0.9)
	Staff	221	177 (79.7)	41 (18.6)	3 (1.4)	0 (0)	0 (0)
	Women	625	480 (76.8)	126 (20.2)	15 (2.4)	0 (0.0)	4 (0.6)
	Men	179	143 (79.9)	30 (16.8)	3 (1.7)	1 (0.6)	2 (1.1)
Do you believe that the food a person eats makes a difference for his or her health in general?	Students	578	420 (72.7)	131 (22.7)	22 (3.8)	0 (0)	5 (0.9)
	Staff	222	173 (77.9)	42 (18.9)	5 (2.3)	1 (0.5)	1 (0.5)
	Women	627	463 (73.8)	137 (21.9)	22 (3.5)	0 (0.0)	5 (0.8)
	Men	178	134 (75.3)	37 (20.8)	5 (2.8)	1 (0.6)	1 (0.6)
Do you believe that smoking negatively affects a person’s health?	Students	578	513 (88.8)	46 (8.0)	11 (1.9)	1 (0.2)	7 (1.2)
	Staff	222	206 (92.8)	12 (5.4)	3 (1.4)	0 (0)	1 (0.5)
	Women	627	573 (91.4)	38 (6.1)	9 (1.4)	1 (0.2)	1 (1.0)
	Men	178	150 (84.3)	21 (11.8)	5 (2.8)	0 (0.0)	2 (1.1)
Do you believe that stress has a negative effect on a person’s health?	Students	572	428 (74.8)	123 (21.5)	10 (1.7)	7 (1.2)	4 (0.7)
	Staff	219	192 (87.7)	21 (9.6)	3 (1.4)	3 (1.4)	0 (0)
	Women	621	493 (79.4)	109 (17.6)	10 (1.6)	7 (1.1)	2 (0.3)
	Men	175	131 (74.9)	35 (20.0)	3 (1.7)	4 (2.4)	2 (1.1)

## Appendix A

**Table A1:** Cut points for health parameters.

Variable	Cut Point	Source
Fruit (servings/day)	≥3/day	Promoting a Healthy Diet for the WHO Eastern Mediterranean Region. http://www.who.int/nutrition/publications/nutrientrequirements/healtydietguide2012_emro/en/
Vegetables (servings/day)	≥6/day	Promoting a Healthy Diet for the WHO Eastern Mediterranean Region. http://www.who.int/nutrition/publications/nutrientrequirements/healtydietguide2012_emro/en/
Red meat (servings/wk.)	≤1x/wk.	Promoting a Healthy Diet for the WHO Eastern Mediterranean Region. http://www.who.int/nutrition/publications/nutrientrequirements/healtydietguide2012_emro/en/
Fish (servings/wk.)	≥2	Promoting a Healthy Diet for the WHO Eastern Mediterranean Region. http://www.who.int/nutrition/publications/nutrientrequirements/healtydietguide2012_emro/en/
Eggs (number/wk.)	≤4	Harvard School of Public Health. Eggs and Heart Disease; http://www.hsph.harvard.edu/nutritionsource/eggs/ Note: although an egg a day is considered acceptable for cardiovascular health, this depends on the size of the egg
Fast food (times/wk.)	Ideally none; little nutritional value	USDA Scientific Report of the 2015 Dietary Guidelines Advisory Committee; http://www.health.gov/dietaryguidelines/2015-scientific-report/PDFs/Scientific-Report-of-the-2015-Dietary-Guidelines-Advisory-Committee.pdf
Sweet consumption (servings/wk.)	≤3	Promoting a Healthy Diet for the WHO Eastern Mediterranean Region. http://www.who.int/nutrition/publications/nutrientrequirements/healtydietguide2012_emro/en/
Soft drinks (cans/wk.)	Ideally none	Promoting a Healthy Diet for the WHO Eastern Mediterranean Region. http://www.who.int/nutrition/publications/nutrientrequirements/healtydietguide2012_emro/en/
Added salt	Minimal or < 1.5 g/day	Promoting a Healthy Diet for the WHO Eastern Mediterranean Region. http://www.who.int/nutrition/publications/nutrientrequirements/healtydietguide2012_emro/en/ American Heart Association and American Stroke Association, Stroke, 2014; http://stroke.ahajournals.org/content/early/2014/04/30/STR.0000000000000024.full.pdf+html (most Na is consumed in processed foods; thus, minimal added salt is required)
Daily breakfast	Yes	O’Neil et al., J Acad Nutr Diet. 2014 Dec; 114(12 Suppl):S27–43.
Physical activity/exercise	2.5 h moderately intense activity orequivalent/w.	Global Strategy on Diet, Physical Activity and Health. Global recommendations on physical activity for health. http:/www.who/dietphysicalactivity/factsheet_recommendations/en/ 2008 Physical Activity Guidelines for Americans. http:/www.health.gov/paguidelines
Stress	Mostly low manageable stress	Harvard Medical School Special Health Report. Positive Psychology. 2011.
Sleep	≥8 h	Harvard Medical School Special Health Report. Improving Sleep. 2013. Coren S. Physiotherapy Theory and Practice, 2009, 25(5):442–452.
Quality of life (general life satisfaction over the past month)	≥7	Visual analogue scale (0 to 10; 0 extremely low and 10 extremely high)
Waist/hip ratio Men Women	0.85 0.75	Yusuf et al. Lancet. 2004; 364(9438):937–952. Yusuf et al. Lancet. 2005; 366(9497):1640–1649.
Waist/height ratio	<0.5	Browning et al. Nutritional Research Review. 2010; 23:247–269.
Body mass index (kg·m^−2^)	18.5 to 24.9	World Health Organization. Global Health Observatory Data. Mean body mass index. http:/www.who.int/gho/ncd/risk_factors/bmi_text/en/
Heart rate (bpm)	60–80	http://www.heart.org/HEARTORG/Conditions/HighBloodPressure/AboutHighBloodPressure/Blood-Pressure-vs-Heart-Rate_UCM_301804_Article.jsp
Resting systolic blood pressure (mm Hg)	≤120	http://www.heart.org/HEARTORG/Conditions/HighBloodPressure/AboutHighBloodPressure/Blood-Pressure-vs-Heart-Rate_UCM_301804_Article.jsp
Resting diastolic blood pressure (mm Hg)	≤80	http://www.heart.org/HEARTORG/Conditions/HighBloodPressure/AboutHighBloodPressure/Blood-Pressure-vs-Heart-Rate_UCM_301804_Article.jsp
Random blood glucose (mmol/L)	>11.1	American Diabetes Association. 2015. Diagnosing Diabetes and Learning About Prediabetes. http://www.diabetes.org/are-you-at-risk/prediabetes/
